# Modelling non-homogeneous stochastic reaction-diffusion systems: the case study of gemcitabine-treated non-small cell lung cancer growth

**DOI:** 10.1186/1471-2105-13-S14-S14

**Published:** 2012-09-07

**Authors:** Paola Lecca, Daniele Morpurgo

**Affiliations:** 1 The Microsoft Research - University of Trento Centre for Computational and Systems Biology, Rovereto, Italy

## Abstract

**Background:**

Reaction-diffusion based models have been widely used in the literature for modeling the growth of solid tumors. Many of the current models treat both diffusion/consumption of nutrients and cell proliferation. The majority of these models use classical transport/mass conservation equations for describing the distribution of molecular species in tumor spheroids, and the Fick's law for describing the flux of uncharged molecules (i.e oxygen, glucose). Commonly, the equations for the cell movement and proliferation are first order differential equations describing the rate of change of the velocity of the cells with respect to the spatial coordinates as a function of the nutrient's gradient. Several modifications of these equations have been developed in the last decade to explicitly indicate that the tumor includes cells, interstitial fluids and extracellular matrix: these variants provided a model of tumor as a multiphase material with these as the different phases. Most of the current reaction-diffusion tumor models are deterministic and do not model the diffusion as a local state-dependent process in a non-homogeneous medium at the micro- and meso-scale of the intra- and inter-cellular processes, respectively. Furthermore, a stochastic reaction-diffusion model in which diffusive transport of the molecular species of nutrients and chemotherapy drugs as well as the interactions of the tumor cells with these species is a novel approach. The application of this approach to he scase of non-small cell lung cancer treated with gemcitabine is also novel.

**Methods:**

We present a stochastic reaction-diffusion model of non-small cell lung cancer growth in the specification formalism of the tool Redi, we recently developed for simulating reaction-diffusion systems. We also describe how a spatial gradient of nutrients and oncological drugs affects the tumor progression. Our model is based on a generalization of the Fick's first diffusion law that allows to model diffusive transport in non-homogeneous media. The diffusion coefficient is explicitly expressed as a function depending on the local conditions of the medium, such as the concentration of molecular species, the viscosity of the medium and the temperature. We incorporated this generalized law in a reaction-based stochastic simulation framework implementing an efficient version of Gillespie algorithm for modeling the dynamics of the interactions between tumor cell, nutrients and gemcitabine in a spatial domain expressing a nutrient and drug concentration gradient.

**Results:**

Using the mathematical framework of model we simulated the spatial growth of a 2D spheroidal tumor model in response to a treatment with gemcitabine and a dynamic gradient of oxygen and glucose. The parameters of the model have been taken from recet literature and also inferred from real tumor shrinkage curves measured in patients suffering from non-small cell lung cancer. The simulations qualitatively reproduce the time evolution of the morphologies of these tumors as well as the morphological patterns follow the growth curves observed in patients.

**Conclusions:**

s This model is able to reproduce the observed increment/decrement of tumor size in response to the pharmacological treatment with gemcitabine. The formal specification of the model in Redi can be easily extended in an incremental way to include other relevant biophysical processes, such as local extracellular matrix remodelling, active cell migration and traction, and reshaping of host tissue vasculature, in order to be even more relevant to support the experimental investigation of cancer.

## Background

As the name indicates, reaction-diffusion models consist of two components. For systems of molecules and atoms, the first component is a set of biochemical reactions which produce, transform or remove chemical species. The second component is a mathematical description of the diffusion process. At molecular level, diffusion is due to the motion of the molecules in a medium. If solutions of different concentrations are brought into contact with each other, the solute molecules tend to flow from regions of higher concentration to regions of lower concentration, and there is ultimately an equalization of concentration. The conceptual framework of a micro-scale reaction-diffusion system can be also adopted to describe the phenomenology of cellular proliferation in tumor growth. Indeed, reaction-diffusion equations based models have been widely used in the literature for modeling the tumor growth. A comprehensive review of reaction-diffusion models and spatial dynamics of tumor growth can be found in [1-4], while specific literature about different variant of reaction-diffusion models of tumor growth can be found in [2,5-13]. Recently, there have been also interesting approaches to the adaptation of general reaction-diffusion models to the specific patient [14,15].

In this application domain, the reaction-diffusion models describe the evolution of the tumors via proliferation of malignant cells and their infiltration into the surrounding healthy tissue (see [16] for a review). The building block of these models is the reaction-diffusion type partial differential equations expressing the rate of change of the tumor cell density as sum of two terms. These terms correspond to the two phenomena described by the model: the diffusion term models, via the first Fick's law, the migration of tumor cells within the tissue and the reaction term, that is polynomial in the tumor cell density, models the proliferation of tumor cells [15]. Different reaction-diffusion models proposed in the literature mostly differ by the construction of the diffusion tensor in the mathematical expression of the Fick's law and the form of the proliferation term [15].

In this study we present a multiscale reaction-diffusion model linking the proliferation of malignant cells to (i) the upshot of the interactions between the oncological drug and the tumor cell, (ii) the availability and the rate of uptake of nutrient by the tumor cells, (iii) and, finally the availability and the rate of consumption of oxygen. Moreover, unlike the majority of the existing models of tumor growth, our model is stochastic, i.e. the interactions between tumor cells and drugs, as well as the events of uptake and consumption of nutrients and oxygen are stochastic Markov events. All the reaction-diffusion events are parallel and concurrent. The probability of a given event to be executed is proportional to the number of substrate molecules (for biochemical reactions) or to the number of cells (for interactions like cell proliferation and tumor growth). Recent studies [17] support this approach and show how the competitive intra-cellular reactions for the uptake and consumption of nutrients and oxygen are crucial in determining the tumor morphology.

We developed a generalization of the Fick's laws to model diffusion of drugs, nutrients and oxygen in the tissue, whereas we use the standard Fick's law to model the tumor cell proliferation and invasion following the gradient of nutrients and oxygen. Namely, the number of tumor cells and their spatial proliferation depend on the diffusion of nutrients, oxygen and drugs through the space and on the results of the interaction of these cell with the anticancer drug.

Before proceeding with a detailed explanation of our model of reaction-diffusion system, we briefly introduce the motivations and the guidelines of our work.

The tumor size provides a measure that is useful for describing the time course of tumor response to the chemotherapic treatment. However, tumor growth changes can be observed only through repeat following-up visits and may require sophisticated and expensive hardware and software imaging techniques especially for monitoring the size of in deep-seated tumors. Due to this reason, the measurements of tumor progression in time and space have yet to gain wide application as an end point for drug effects modelling in clinical trials. The measurements of tumor size are still principally used for tumor stage categorization, whereas in the early-phase clinical trials the measurements of changes in hematologic variables have been used as pharmacodynamic targets [18]. Therefore, a pharmacodynamic model that describes the interactions of tumor cells with the oncological drug and with nutrients, as well as the drug effects may have practical potential as a midterm end point for decision making about drug administration schedule and treatment duration. In this article, we focus on the modelling and simulation of the growth of non-small cell lung cancer treated with gemcitabine. This kind of cancer and its pharmacological treatment have been recently studied and new experimental data concerning both the measurements of the progression of tumor size [18] and the pharmacokinetics and pharmacodynamics of the gemcitabine [19] are now available and enable us to use of the computational models presented in this study.

In order to build an accurate and predictive model of tumor growth, the physical and biochemical non-homogeneous environments in which the tumor arises and progresses, a generalization of the current mathematical formalization of reaction-diffusion systems is needed both at the micro-scale of the intra-cellular phenomena and at the meso-scale of inter-cellular and tissue processes. A preponderance of reaction-diffusion models of intra- and inter- cellular kinetics is usually performed on the premise that diffusion is so fast that all concentrations are maintained homogeneous in space. However, recent experimental data on intra- and inter-cellular diffusion constants, indicate that this supposition is not necessarily valid even for small prokaryotic cells [20,21]. If the system is composed of a sufficiently large number of molecules, the concentration, i. e. the number of molecules per unit volume, can be represented as a continuous and differentiable variable of space and time. In this limit a reaction diffusion system can be modeled using differential equations. In an unstructured solvent, ideally behaving solutes (i.e. solutes for which solute-solute interaction are negligible) obey the Fick's law of diffusion. However in biological systems even for purely diffusive transport phenomena classical Fickian diffusion is, at best, a first approximation [22,23]. The intra- and the inter-cellular media are not homogeneous mixtures of chemical species, but highly structured environments partitioned into compartments in which the distribution of the biomolecules could be non-homogeneous. The description of diffusion processes in this environment has to start from a model in which diffusion coefficient contains its dependency on the local concentrations of the solutes and solvent.

In order to tackle this problem, this paper presents a new model of diffusion coefficient for a non-homogeneous non-well-stirred reaction-diffusion system. In this model the diffusion coefficient explicitly depends on the local concentration, frictional coefficient of the particles of the systems, and of the temperature of the reaction environment. In turn, the rates of diffusion of the biochemical species are expressed in terms of these concentration-dependent diffusion coefficients. In this study the purely diffusive transport phenomena of non-charged particles, and, in particular, the case in which diffusion is driven by a chemical potential gradient in the *x *direction only (the generalization to the three-dimensional case easily ensues) are considered. The generalization of the Fick's law introduced in this work, consists of five main steps: 1. calculation of the *local *virtual force *F *per molecules as the spatial derivative of the chemical potential; 2. calculation of the particles mean drift velocity in terms of *F *and the local frictional coefficient *f*; 3. estimation of the flux *J *as the product of the mean drift velocity and the local concentration; 4. definition of diffusion coefficients as function of local activity and frictional coefficients and concentration; and 5. calculation of diffusion rates as the negative first spatial derivative of the flux *J*.

The diffusion events are modeled as reaction events and the spatial domain of the reaction chamber is divided into *N_s _*sub-volumes (or meshes) of size *l*, that from now on will be called *meshes*. The movement of a molecule *A *from box *i *to box *j *is represented by the reaction $A_{i}\overset{k}{\to }A_{j}$, where *A_i _*denotes the molecule *A *in the box *i *and *A_j _*denotes the molecule *A *in the box *j*. The reaction-diffusion system is thus modeled as a pure reaction system in which the diffusion events are first order reactions whose rate coefficients *k *are expressed in terms of state-dependent diffusion coefficients. The time evolution of the system is computed by a Gillespie-like algorithm [24] that selects at each simulation step in each mesh the fastest reaction, compares the velocities of the *N_s _*selected reactions and finally executes the reaction that is by far the fastest.

The paper outlines as follows. First we describe our generalization of the Fick's law, then we briefly describe how it can be incorporated in a stochastic simulation framework. Finally, we present a model of tumor growth and the simulation results.

## Methods

We summarize here the main passages of the generalization of the Fick's first law. We refer the reader to Lecca *et al.*[25] for a more comprehensive description of the mathematical structures and passages.

### A generalization of Fick's law for modeling diffusion

In a chemical system the driving force for diffusion of each species is the gradient of chemical potential *µ *of this species. The chemical potential of any particular chemical species *i *is

(1) $$\mu_{i}=\mu_{i}^{0}+RT\text{ln}a_{i},$$

where $\mu_{i}^{0}$ is the standard chemical potential of the species *i *(i.e. the Gibbs energy of 1 mol of species *i *at a pressure of 1 bar), *R *= 8.314 J · K*^-^*^1 ^*· *mol^-1 ^is the ideal gas constant, and *T *the absolute temperature.

The quantity *a_i _*is called *chemical activity *of component *i*, and it is given by

(2) $$a_{i}=\frac{\gamma_{i}c_{i}}{c^{0}}$$

where *γ_i _*is the *activity coefficient*, and *c*^0 ^a reference concentration, which, for example, could be set equal to the initial concentration. The activity coefficients express the deviation of a solution from ideal thermodynamic behavior and, in general, they may depend on the concentration of all the solutes in the system. For an ideal solution, the limit of *γ_i_*, which is recovered experimentally at high dilutions is *γ_i _*= 1. If the concentration of species *i *varies from point to point in space, then so does the chemical potential. For simplicity, here the case in which there is only a chemical potential gradient in the *x *direction is taken into account. Chemical potential is the free energy per mole of substance, free energy is the negative of the work, *W*, which a system can perform, and work is connected to force *F *acting on the molecules by *dW *= *Fdx*. Therefore an inhomogeneous chemical potential is related to a virtual force per molecule of

(3) $$F_{i}=-\frac{1}{N_{A}}\frac{d\mu_{i}}{dx}=-\frac{k_{B}Tc^{0}}{\gamma_{i}c_{i}}\underset{j}{\sum }\frac{\partial a_{i}}{\partial c_{j}}\frac{\partial c_{j}}{\partial x}$$

where *N_A _*= 6.022 × 10^23^ mol^-1^ is Avogadro's number, *k_B _*= 1.381 × 10^-23^ J · K^-1 ^is the Boltzmann constant, and the sum is taken over all species in the system other than the solvent. This force is balanced by the drag force experienced by the solute (*F_drag_*, *_i_*) as it moves through the solvent. Drag forces are proportional to speed. If the speed of the solute is not too high in such a way that the solvent does not exhibit turbulence, the drag force can be written as follows

(4) $$F_{drag,i}=f_{i}v_{i},$$

where *f_i _*α *c_i _*is the frictional coefficient, and *v_i _*is the mean drift speed.

Moreover, if the solvent is not turbulent, the *flux*, defined as the number of moles of solute which pass through a small surface per unit time per unit area, can be approximated as in the following

(5) $$J_{i}=c_{i}v_{i},$$

i.e. the number of molecules per unit volume multiplied by the linear distance traveled per unit time.

Since the virtual force on the solute is balanced by the drag force (i. e. *F_drag,i _*= *-F_i_*), the following expression for the mean drift velocity is obtained

$$v_{i}=\frac{F_{i}}{f_{i}},$$

so that Eq. (5) becomes

(6) $$J_{i}=-\frac{k_{B}T}{\gamma_{i}f_{i}}\underset{j}{\sum }\frac{\partial a_{i}}{\partial c_{j}}\frac{\partial c_{j}}{\partial x}=-\underset{j}{\sum }D_{ij}\frac{\partial c_{j}}{\partial x},$$

where

(7) $$D_{ij}=\frac{k_{B}Tc^{0}}{\gamma_{i}f_{i}}\frac{\partial a_{i}}{\partial c_{j}},$$

are the diffusion coefficients. The Eq. (7) states that, in general, the flux of one species depends on the gradients of all the others, and not only on its own gradient. However, here it is supposed that the chemical activity *a_i _*depends only weakly on the concentrations of the other solutes, i.e. it is assumed that *D_ij _*≈ 0 for *i *≠ *j *and that Fick's laws still holds [26]. Let *D_i _*denote *D_ii_*. It is still generally the case that *D_i _*depends on *c_i _*in sufficiently concentrated solutions since *γ_i _*(and thus *a_i_*) has a non trivial dependence on *c_i _*[26]. There is only one very special case, namely that of an ideal solution with *γ_i _*= 1, in which the diffusion coefficient, *D_i _*= *k_B_T/f_i_*, is constant. In order to find an analytic expression for the diffusion coefficient, *D_i_*, in terms of the concentration, *c_i_*, let us consider that the rate of change of concentration of the substance *i *due to diffusion is given by

(8) $$D_{i}=-\frac{\partial J_{i}}{\partial x},$$

Substituting Eq. (7) into Eq. (6), and then substituting the obtained expression for *J_i _*into Eq. (8), give

$$D_{i}=-\frac{\partial }{\partial x}\left(-D_{i}\left(c_{i}\right)\frac{\partial c_{i}}{\partial x}\right),$$

so that

$$D_{i}=\left(\frac{\partial D_{i}\left(c_{i}\right)}{\partial x}\right)\frac{\partial c_{i}}{\partial x}+D_{i}\left(c_{i}\right)\frac{\partial^{2}c_{i}}{\partial x^{2}}=\frac{\partial D_{i}\left(c_{i}\right)}{\partial c_{j}}\frac{\partial c_{j}}{\partial x}\frac{\partial c_{i}}{\partial x}+D_{i}\left(c_{i}\right)\frac{\partial^{2}c_{i}}{\partial x^{2}}.$$

Let *c_i_,_k _*denote the concentration of a substance *i *at coordinate *x_k_*, and *l *= *x_k _*- *x_k_*_-1 _the distance between adjacent mesh points. The derivative of *c_i _*with respect to *x *calculated at $x_{k-\frac{1}{2}}$ is

(9) $${\left(\frac{\partial c_{i}}{\partial x}\right|}_{x_{k-\frac{1}{2}}}\approx \frac{{c_{i}}_{,k}-{c_{i}{}_{,k}}_{-1}}{l}.$$

By using Eq. (9) into Eq. (6) the diffusive flux of species *i *midway between the mesh points, $J_{i,k-\frac{1}{2}}$ is obtained:

(10) $$J_{i,k-\frac{1}{2}}=-D_{i,k-\frac{1}{2}}\frac{c_{i,k}-c_{i,k-1}}{l},$$

where $D_{i,k-\frac{1}{2}}$ is the diffusion coefficient midway between the mesh points.

The rate of diffusion of substance *i *at mesh point *k *is

$$D_{ik}=-\frac{J_{i,k+\frac{1}{2}}-J_{i,k-\frac{1}{2}}}{l},$$

and thence

(11) $$D_{ik}=\frac{D_{i,k-\frac{1}{2}}}{l^{2}}\left(c_{i,k-1}-c_{i,k}\right),-\frac{D_{i,k+\frac{1}{2}}}{l^{2}}\left(c_{i,k+1}-c_{i,k}\right)$$

To determine completely the right-hand side of Eq. (11) is now necessary to find an expression for the activity coefficient, *γ_i_*, and the frictional coefficient, *f_i_*, contained in the expression of the diffusion coefficient. In fact, by substituting Eq. (2) into Eq. (7) we obtain the diffusion coefficient in terms of activity coefficients *γ_i_*:

(12) $$D_{ii}=\frac{k_{B}T}{f_{i}}\left(1+\frac{c_{i}}{\gamma_{i}}\frac{\partial \gamma_{i}}{\partial c_{i}}\right)$$

where the frictional coefficient is assumed to be linearly dependent on the concentration of the solute like in sedimentation processes, i.e. in a mesh *k*, *fi*, *k *is

(13) $$f_{i,k}=k_{f}c_{i,k}$$

where *k_f _*is an empirical constant, whose value can be derived from the ratio *R *= *k_f_/*[*η *]: Accordingly to the Mark-Houwink equation [27], [*η *] = *kM^α ^*is the intrinsic viscosity coefficient, *α *is related to the shape of the molecules of the solvent, and *M *is the molecular mass of the solute. If the molecules are spherical, the intrinsic viscosity is independent of the size of the molecules, so that *α *= 0. All globular proteins, regardless of their size, have essentially the same [*η *]. If a protein is elongated, its molecules are more effective in increasing the viscosity and [*η *] is larger. Values of 1.3 or higher are frequently obtained for molecules that exist in solution as extended chains. Long-chain molecules that are coiled in solution give intermediate values of is α, frequently in the range from 0.6 to 0.75 [27]. For globular macromolecules, *R *has a value in the range of 1.4 - 1.7, with lower values for more asymmetric particles [28].

Although Eq. (13) is a simplified linear model of the frictional forces, it works quite well in many case studies and can be easily extended to treat more complex frictional effects (see [25,29]).

Let us focus now on calculation of the activity coefficients: a way to estimate the frictional coefficients will be presented in the next subsection. By using the subscript '1' to denote the solvent and '2' to denote the solute, it can be written that

(14) $$\mu_{2}=\mu_{2}^{0}+RT\text{ln}\left(\frac{\gamma_{2}c_{2}}{c^{0}}\right),$$

where *γ*_2_ is the activity coefficient of the solute and *c*_2_ is the concentration of the solute. Differentiating with respect to *c*_2_ gives

(15) $$\frac{\partial \mu_{2}}{\partial c_{2}}=RT\left(\frac{1}{c_{2}}+\frac{1}{\gamma_{2}}\frac{\partial \gamma_{2}}{\partial c_{2}}\right).$$

The chemical potential of the solvent is related to the osmotic pressure (II) by

(16) $$\mu_{1}=\mu_{1}^{0}-\Pi V_{1},$$

where *V*_1_ is the partial molar volume of the solvent and $\mu_{1}^{0}$its standard chemical potential. Assuming *V*_1_ to be constant [30] and Differentiating *μ*_1_ with respect to *c*_2_ yield

(17) $$\frac{\partial \mu_{1}}{\partial c_{2}}=-V_{1}\frac{\partial \Pi }{\partial c_{2}}$$

Now, from the Gibbs-Duhem relation [31], the derivative of the chemical potential of the solute with respect to the solute concentration is

(18) $$\frac{\partial \mu_{2}}{\partial c_{2}}=-\frac{M\left(1-c_{2}\bar{v}\right)}{V_{1}c_{2}}\frac{\partial \mu_{1}}{\partial c_{2}}=\frac{M\left(1-c_{2}\bar{v}\right)}{c_{2}}\frac{\partial \Pi }{\partial c_{2}},$$

where *M *is molecular mass of the solute and $\bar{v}$ is the partial molar volume of the solute divided by its molecular mass. The concentration dependence of osmotic pressure is usually written as

(19) $$\frac{\Pi }{c_{2}}=\frac{RT}{M}\left[1+BMc_{2}+O\left(c_{2}^{2}\right)\right].$$

where *B *is the second virial coefficient, and thence the derivative of II with respect to the solute concentration is

(20) $$\frac{\partial \Pi }{\partial c_{2}}=\frac{RT}{M}+2RTBc_{2}+O\left(c_{2}^{2}\right).$$

Introducing Eq. (20) into Eq. (18) gives

(21) $$\frac{\partial \mu_{2}}{\partial c_{2}}=RT\left(1-c_{2}\bar{v}\right)\;\left(\frac{1}{c_{2}}+2BM\right).$$

From Eq. (15) and Eq. (21) it can be obtained that

$$\frac{1}{\gamma_{2}}\frac{\partial \gamma_{2}}{\partial c_{2}}=\frac{1}{c_{2}}\left[\left(1-c_{2}\bar{v}\right)\left(1+2BMc_{2}\right)-1\right],$$

so that

$$\int_{1}^{{\gamma^{\prime }}_{2}}\frac{d\gamma_{2}}{\gamma_{2}}=\int_{c^{0}}^{{c^{\prime }}_{2}}\frac{1}{c_{2}}\left[\left(1-c_{2}\bar{v}\right)\left(1+2BMc_{2}\right)-1\right]dc_{2}.$$

On the grounds that $c_{2}\bar{v}\ll 1$[32], solving the integral yields

(22) $$\gamma_{2}^{\prime }=\text{exp}\left[2BM\left(c_{2}^{\prime }-c^{0}\right)\right]$$

The molecular mass *M_i,k _*of the species *i *in the mesh *k *can be expressed as the ratio between the mass, *m_i,k_*, of the species *i *in that mesh and the Avogadro number *M_i,k _*= *m_i,k _/N_A_*. If *p_i _*is the mass of a molecule of species *i *and *c_i,k _*· *l *is the number of molecules of species *i *in the mesh *k*, then the molecular mass of the solute of species *i *in the mesh *k *is given by

(23) $$M_{i,k}=\frac{p_{i}\;\;l}{N_{A}}c_{i,k}.$$

Substituting the expression in Eq. (22) gives, for the activity coefficient of the solute of species *i *in the mesh *k *(*γ_i;k_*), the following equation

(24) $$\gamma_{i,k}=\text{exp}\left(2B\frac{p_{i}\;\;l}{N_{A}}c_{i,k}^{2}\right).$$

Therefore, substituting Eq. (13) and Eq. (24) into Eq. (12), we obtain the following expression for a time- and space-dependent diffusion coefficient

(25) $$D_{ii}=\frac{k_{B}T}{k_{f}c_{i}}\left(1+\frac{4Bp_{i}\;\;l}{N_{A}}c_{i}\right)$$

We finally estimated in the following way the second virial coefficient *B*. The statistical mechanics definition of the second virial coefficient is as follows

(26) $$B=-2\pi N_{A}\int_{0}^{\infty }r^{2}\text{exp}\left[-\frac{u\left(r\right)}{k_{B}T}\right]dr$$

where *u*(*r*), which is given in Eq. (27), is the interaction free energy between two molecules, *r *is the intermolecular center-center distance, *k_B _*is the Boltzman constant, and *T *the temperature. In this work, it is assumed that *u*(*r*) is the Lennard-Jones pair (12,6)-potential (Eq. 27), that captures the attractive nature of the Van der Waals interactions and the very short-range Born repulsion due to the overlap of the electron clouds:

(27) $$u\left(r\right)=4\left[{\left(\frac{1}{r}\right)}^{12}-{\left(\frac{1}{r}\right)}^{6}\right].$$

By expanding the term exp $\left(\frac{4}{k_{B}T}\;\frac{1}{r^{6}}\right)$into an infinite series, the Eq. (26) becomes

$$B=-2\pi N_{A}\overset{\infty }{\underset{j=0}{\sum }}\frac{1}{j!}{\left(T^{*}\right)}^{j}\int_{0}^{\infty }r^{2-6j}\text{exp}\left[-T^{*}\frac{1}{r^{2}}\right]dr,$$

where T* ≡ 4/(*k_B_T *) and thus

(28) $$B=-\frac{\pi N_{A}}{6}\overset{\infty }{\underset{j=0}{\sum }}\frac{1}{!j}4^{j}{\left(k_{B}T\right)}^{-\frac{1}{4}\;+\;\frac{1}{2}j}\Gamma \left(-\frac{1}{4}+\frac{1}{2}j\right)$$

An estimate of *B *is given by truncating the infinite series of functions to *j *= 4, since simulation results not shown here prove that taking into account the additional terms, obtained for *j >*4, does not significantly influence the simulation results [25].

### Modelling the stochasticity

Both diffusion and reactions are modelled as reaction events whose dynamics is driven by the First Reaction Method of the Gillespie algorithm.

In particular, the diffusion events are modeled as first-order reactions. namely, the movement of a molecule *A *from box *i *to box *j *is represented by the reaction $A_{i}\overset{k}{\to }A_{j}$, where *A_i _*denotes the molecule *A *in the mesh *i *and *A_j _*denotes the molecule *A *in the mesh *j*. In this way, the reaction-diffusion system is modeled as a pure reaction system.

The space domain of the system is divided into a number *N_s _*of squared meshes of size *l*. The time evolution of the system is computed by the First reaction Method of Gillespie [24] that at each simulation step selects in each mesh the fastest reaction, compares the velocities of the *N_s _*selected reactions and finally executes the reaction that is by far the fastest. The fastest reaction is defined as the reaction whose waiting time is the smallest.

The time at which each event is expected to occur is a random variable extracted by an exponential distribution [24]. Let *R_i _*be the i-*th *reaction channel expressed as

$$R_{i}:l_{i1}S_{p\left(i,1\right)}+l_{i2}S_{p\left(i,2\right)}+\cdots +l_{iL_{i}}S_{p\left(i,L_{i}\right)}\overset{r_{i}}{\to }\;\text{. }\text{. }.$$

where *l_ij _*is the stoichiometric coefficient of reactant *S_p_*(*_i,j_*), *p*(*i*, *j*) is the index that selects the species that participates in *R_i_*, *L_i _*is the number of reactants in *R_i_*, and *r_i _*is the rate constant. If the fundamental hypothesis of stochastic chemical kinetics [24] holds within a box, both diffusion and reaction events waiting times are distributed according to a negative exponential distribution, so that a typical time step has size

(29) $$t_{r}\approx \frac{1}{R}{\left(\overset{R}{\underset{\nu =1}{\sum }}a_{\nu }\right)}^{-1}=\frac{1}{R}\left(\overset{R_{diff}}{\underset{i=1}{\sum }}a_{i}^{\left(diff\right)}+\overset{R_{r\text{e}act}}{\underset{i=1}{\sum }}a_{i}^{\left(react\right)}\right)$$

where *R *is the number of events. It is given by *R *= *R_diff _*+ *R_react_*, where *R_diff _*is the number of possible diffusion events and *R_react _*is the number of reaction events [33]. The diffusion and reaction propensities are given by the following expressions, respectively

(30) $$a_{i}^{\left(diff\right)}=r_{i}^{\left(diff\right)}\frac{{\prod^{\text{​}}}_{j=1}^{M_{i}^{\left(diff\right)}}({\left[S_{p(i,j)}\right)}^{l_{ij}}}{{\prod^{\text{​}}}_{j=1}^{L_{i}^{\left(diff\right)}}l_{ij}!},$$

(31) $$a_{i}^{\left(react\right)}=r_{i}^{\left(react\right)}\frac{{\prod^{\text{​}}}_{j=1}^{M_{i}^{\left(react\right)}}({\left[S_{p(i,j)}\right)}^{l_{ij}}}{{\prod^{\text{​}}}_{j=1}^{L_{i}^{\left(react\right)}}l_{ij}!},$$

where $M_{i}^{\left(diff\right)}$ and $M_{i}^{\left(react\right)}$ are the number of chemical species that diffuse and the number of those the undergo to reactions, respectively. In general $M\;\neq \;M_{i}^{\left(diff\right)\;}+\;M_{i}^{\left(react\right)}$, since some species both diffuse and react. In Eq. (30), *r_i_*^(*diff*) ^is the kinetic rate associated to the jumps between neighboring subvolumes, whereas in Eq. (31), *r_i_*^(*react*) ^is the stochastic rate constants of the i-*th *reaction.

From Eq. (11), the rate coefficient of the first order reaction representing a diffusion event is recognized to be as follows

(32) $$r_{i}^{\left(diff\right)}=\frac{D_{ii}}{l^{2}}.$$

## Results

Here we describe the model of tumor growth implemented with the toll Redi. Redi is a software prototype *Redi *[25] that has been recently developed by Lecca et al. [25,34] to simulate the mathematical model of stochastic reaction-diffusion system that we have described in the previous section. We refer the reader to the references [25,34-36] for technical details about the implementation and for a user manual of this software.

### Model of tumor growth

The reaction-diffusion system modelling tumor growth involves four components: (i) the drug, gemcitabine, (ii) the tumor cell, (iii) oxygen, and (iv) glucose. The reaction events we modeled are the following:

R1. gemcitabine injection;

R2. gemcitabine diffusion;

R3. gemcitabine degradation (rate parameter *k*_1_);

R4. effective interaction of gemcitabine and death of tumor cell (rate parameter *k*_2_);

R5. ineffective interaction of gemcitabine: the tumor cell survives to the drug (rate parameter *k*_3_);

R6. tumor growth (rate parameter *k*_4_);

R7. glucose uptake (rate parameter *k*_5_);

R8. oxygen uptake (rate parameter *k*_6_);

R9. glucose diffusion;

R10. oxygen diffusion;

R11. tumor turnover (rate parameter *k*_7_).

With regard to the dosage schedule of gemcitabine (event R1), we simulated the administration regime proposed by Tham et al. [18] and Soo et al. [37], i.e. gemcitabine was infused at a fixed dose rate of 1,000 mg/m^2 ^over 30 min on day 1 and 8 every three weeks. The diffusion coefficient of gemcitabine is automatically calculated by Redi as a function of space and time according to the formula (25). The efficacy of the gemcitabine (*k*_2_), the rate constant for resistance appearance (*k*_3_), and the tumor growth rate (*k*_4_) have been inferred with KInfer (a maximum likelihood parameter estimator recently developed by Lecca et al. [38,39]) from the tumor size shrinkage curves observed in 56 patients treated with gemcitabine [18]. The 56 patients have been categorized by their age, sex and smoke history, and in our simulations we considered the average values of *k*_2_, *k*_3_, and *k*_4_ (see in Tables 1 and 2 the average values and the standard deviations of these three parameters).

**Table 1:** values of parameters and variables in the three models.

Variable	Model 1	Model 2	Model 3
Nr. of tumor cell per mesh	2547	100	2457
Amount of gemcitabine per mesh	1 *μg*	1 *μg*	10 μg ^*^
Amount of glucose per mesh	792 *μg*	-	-
Amount of oxygen per mesh	3 *μg*	-	-
Parameter	Model 1	Model 2	Model 3
Gemcitabine infusion rate (*k*_0_)	0.56 pg/sec	-	-
Gemcitabine degradation (*k*_1_)	2.78 × 10^-5^ sec^-1^	-	-
Gemcitabine efficacy (*k*_2_)	8.33 × 10^-7^ (mm · sec) ^-1^	-	-
Rate constant of resistance appearance (*k*_3_)	2.78 × 10^-8^	-	-
Tumor growth rate (*k*_4_)	5.56 × 10^-5^ mm/sec	-	-
Glucose uptake rate constant(*k*_5_)	10.4 pg/sec	0.0104 pg/sec	10.4 pg/sec
Oxygen uptake rate constant (*k*_6_)	0.16 pg/sec	-	-
Tumor turnover (*k*_7_)	218 mm · week	-	-
Molecular weight of gemcitabine	0.29966 kD	-	-
Molecular weight of gslucose	0.18016 kD	-	-
Molecular weight of oxygen	0.01801528 kD	-	-


*k*_0_, *k*_1_, *k*_5_, *k*_6_, and *k*_7 _have been reported from the literature, whereas ***k*_2 _**and ***k*_3 _**has been estimated with KInfer [38] from the tumor growth curve of 56 patients provided by the experiments of Tham et al. [18].

The estimate of ***k*_2 _**and ***k*_3 _**is an average of 56 estimates, and is affected by a standard deviation of 6.23 × 10^-**7**^ (mm **· **sec)^-**1**^. The symbol "- " means that the value of the parameter or variable is unchanged.

^*^ This dose correspond to 10,600 mg body concentration of drug (optimal dose estimated in [18].

**Table 2:** categorization of patients and average values of gemcitabine efficacy.

Category of patient	Median value of efficacy
Male	0.03817219 (cm · hours)^-^1
Female	0.03815441 (cm · hours)^-^1
Smoker	0.02937583 (cm · hours)^-^1
Ex-smoker	0.07753538 (cm · hours)^-^1
Non-Smoker	0.03815441 (cm · hours)^-^1

Finally, the parameters of reactions R7 (*k*_5_) and R8 (*k*_6_) have been taken from [40,41] and [42,43], respectively.

According to reactions R9 and R10, tissues receive glucose and oxygen perfusing through the vessel wall and diffusing in the extracellular space.

Finally, the event R11 (tumor turnover) refers to the replacement of old tumor cells with newly generated ones from the existing ones. Tumor turnover is measured in units of sec · mm, and its value (*k*_7_) for non-small lung cancer cell has been measured by Tham et al. [18].

We simulated the morphological changes of an irregular 2D spheroidal tumor having an initial diameter of 3 mm. The size of the computational space is 40 × 40 squared meshes each of which represents a squared portion of tissue having a side of 1 mm. If we assume that the cells have a diameter of 50 *μ*m, a mesh of 1 mm^2 ^is approximately occupied by 2457 cells.

**Figure 1:**
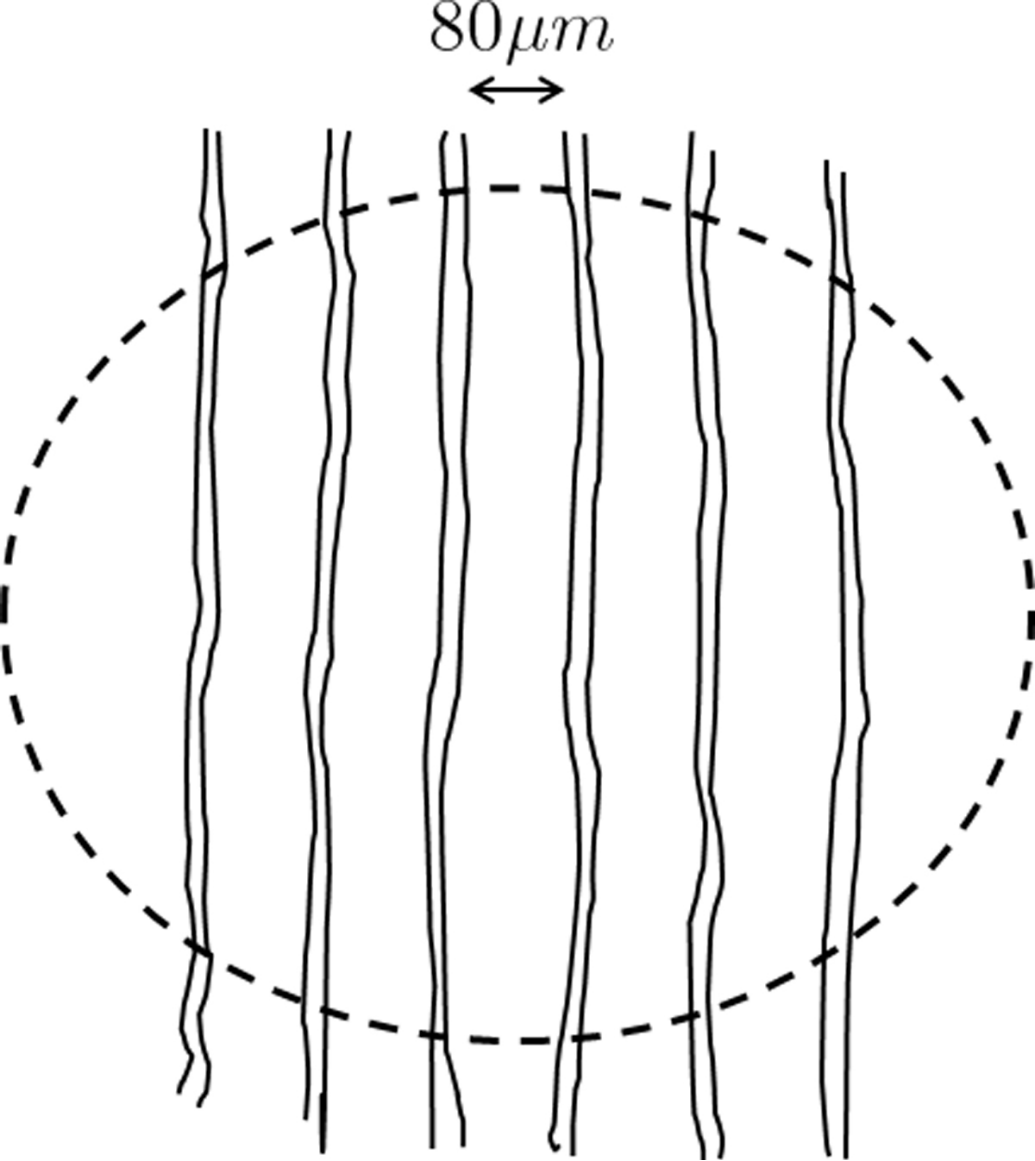
**A simple model of the vascular network innervating the tumor**. The distance between capillaries is 80 *μ*m.

We assumed that the initial spatial distribution of gemcitabine exhibits a gradient pointing outside the tumor. Furthermore we assumed that the tumor as well as the surrounding healthy tissue are crossed by a vascular network of capillaries separated by a distance of 80 *μm *each from the other (see Figure 1). The glucose and oxygen are supplied by the capillaries and they diffuse through the tumor tissue with a rate of diffusion defined by Eq. (11). Their diffusion coefficients are calculated with the formula (25).

All the events R1-R11 are modelled as reaction-events as in the following

R1. gemcitabine injection: zeroth-order reaction ∅ → gemcitabine

R2. gemcitabine diffusion: first order reaction modelling the movement of gemcitabine molecules from mesh *k *to the mesh *k*': gemcitabine*_k _*$\overset{r_{\text{gemcitabine}}^{\left(diff\right)}}{\underset{}{\to }}$ gemcitabine*_k', _*where $r_{\text{gemcitabine}}^{\left(diff\right)}$defined by Eq. (32);

R3. gemcitabine degradation (rate parameter *k*_1_);

R4. effective interaction of gemcitabine and death of tumor cell (rate parameter *k*_2_);

R5. ineffective interaction of gemcitabine: the tumor cell survives to the drug (rate parameter *k*_3_);

R6. tumor growth (rate parameter *k*_4_);

R7. glucose uptake (rate parameter *k*_5_): zeroth-order reaction $\emptyset \overset{k_{5}}{\to }$glucose;

R8. oxygen uptake (rate parameter *k*_6_): : zeroth-order reaction ; $\emptyset \overset{k_{5}}{\to }$ oxygen;

R9. glucose diffusion: first order reaction modelling the movement of gemcitabine molecules from mesh *k *to the mesh *k'*: glucose *_k _*$\overset{r_{\text{glucose}}^{{}^{\left(diff\right)}}}{\underset{}{\to }}$ glucose*_k'_*, where r^(*diff*)^ is defined by Eq. (32);

R10. oxygen diffusion: first order reaction modelling the movement of gemcitabine molecules from mesh *k *to the mesh *k'*: oxygen*_k _*$\overset{r_{\text{oxygen}}^{{}^{\left(diff\right)}}}{\underset{}{\to }}$ oxygen*_k'_*, where r^(*diff*)^ is defined by Eq. (32);

R11. tumor turnover (rate parameter *k*_7_): a zeroth-order reaction describes the generation of new tumor cells from the existing ones; a subsequent first order reaction specifies the replacement of old tumor cells with newly generated ones.

We developed three models, by changing the values of the glucose uptake, the dose of gemcitabine and the number of tumor cell per mesh. In vivo and in vitro experiments carried on in the last decade highlight the crucial role of these variables in governing the dynamics of tumor growth. Some reference experimental studies in this regards are reported in [44-47]. Table 1 reports the initial values of the variables as well as the values of the parameters in the three models. The Model 1 is the reference model whose parameter have physiological values and the dose of gemcitabine is the one usually administered in vivo and in vitro clinical trials. In Model 2 we decreased the number of tumor cells per mesh and the rate of glucose uptake (100 cells per mesh instead of 2547 cells per mesh). In Model 3, we increased the dose of gemcitabine (10 *μg *instead of 1 *μg*). The orders of magnitude of changes of the parameter values in Model 2 and Model 3 are those that cause a significant change in the tumor growth progression.

**Figure 2:**
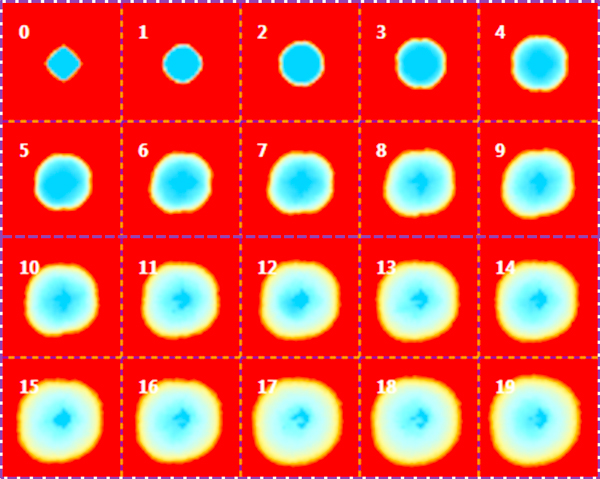
**Simulation of Model 1**. The time unit is the week. The time separating a screenshot from the previous one is 10 weeks. The parameters of the model are listed in Table 1. The longitudinal initial size of the tumor spheroid is 3 mm. Screenshot number "0" is the state of the tumor after 10 weeks of treatment. In the spatial domain of tumor lesion each mesh hosts only tumor cells2. Blue regions are those occupied by more that 2000 tumor cells, yellow regions corresponds to areas of tissue with a number of tumor cells between 100 and 2000, and orange regions are those occupied by less that 100 tumor cells. The extension of the tumor increases linearly in time.

**Figure 3:**
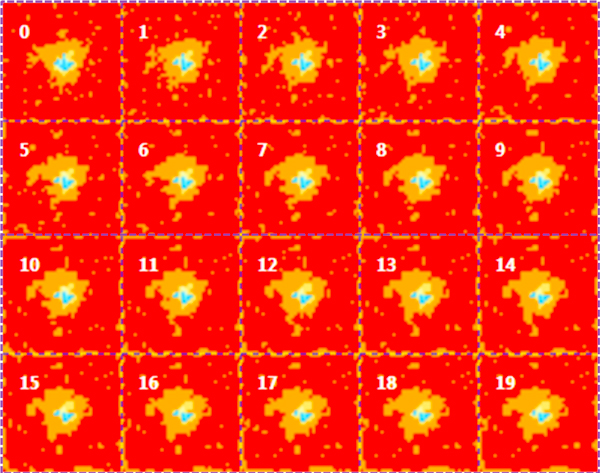
**Simulation of Model 2**. The time unit is the week. The time separating a screenshot from the previous one is 10 weeks. The parameters of the model are listed in Table 1. The initial diameter of the tumor ellipsoid is 3 mm. Screenshot number "0" is the state of the tumor after 10 weeks of treatment. In this model, in the spatial domain of tumor lesion, a mesh hosts both healthy and tumor cells. The number of tumor cells is 100 per mesh and the rate of glucose uptake is two order of magnitude smaller than in rate of glucose uptake in Model 1. As in Figure 1, blue regions are those occupied by more that 2000 tumor cells, yellow regions corresponds to areas of tissue with a number of tumor cells between 100 and 2000, and orange regions are those occupied by less that 100 tumor cells. The size of the tumor is approximately constant, but filaments of tumor cells propagate from the border of the tumor.

**Figure 4:**
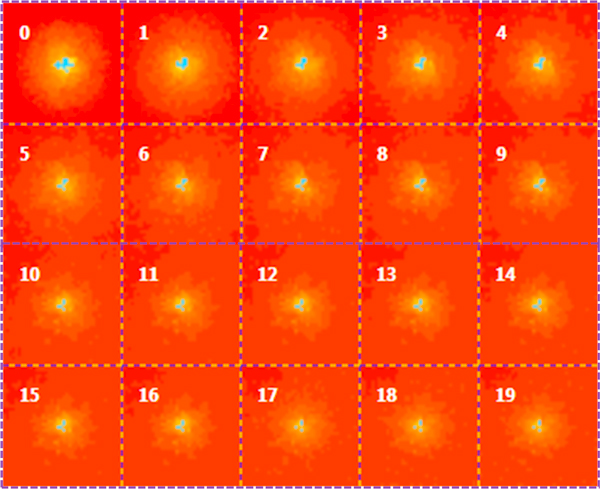
**Simulation of Model 3**. The time unit is the week. The parameters of the model are listed in Table 1. The initial diameter of the tumor ellipsoid is 3 mm. Screenshot number "0" is the state of the tumor after 10 weeks of treatment. In this model, in the spatial domain of tumor lesion, a mesh hosts both healthy and tumor cells. The number of tumor cells per mesh is 2457 as in Model 1 and the rate of glucose uptake is two order of magnitude smaller than the rate of glucose uptake in Model 1. As in Figure 1 and Figure 2, blue regions are those occupied by more that 2000 tumor cells, yellow regions corresponds to areas of tissue with a number of tumor cells between 100 and 2000, and orange regions are those occupied by less that 100 tumor cells. The size of the tumor is approximately constant, but filaments of tumor cells propagate from the border of the tumor, but are disrupted by the action of gemcitabine.

The average of 100 simulations for each model (Model 1, 2 and 3) is showed in Figures 2, 3, and 4 respectively. Each sub-figure is a screenshot of the state of the tumor size recorded each 10 weeks. Blue regions corresponds to areas occupied by more that 2000 tumor cells, yellow regions corresponds to areas of tissue with a number of tumor cell between 100 and 2000, and orange regions are those occupied by less that 100 tumor cells. The simulation of Model 1 in Figure 2 shows a progressive quasi-linear growth of the tumor size at a rate of about 0.5 mm per week. The simulation of Model 2 shows that with a lower rate of glucose uptake the growth of the tumor is slowed down and the borders of the tumor ellipsoid are strongly irregular. Moreover, groups of cells originally belonging to the borders of the tumor proliferate in filaments in healthy parts of the tissue. The action of gemcitabine breaks these filaments but some tumor cells of the filaments still persist in isolated groups infiltrated into the healthy tissue.

Figure 3 shows the simulation of Model 3, where we increased the dose of gemcitabine by a factor 10 to explore the behavior of the tumor mass for the extreme limit of an unrealistic dosage configuration. The rate of glucose uptake is the same as in Model 1. We observed a behavior similar to the one observed in the simulation of Model 1.

As expected, from these simulations we deduced that the effect of gemcitabine is stronger (i) at the early stage of the tumor (i.e. when the number of tumor cells is still low) and the rate of glucose uptake is also low (Model 2), or (ii) if the dose if greater them 1,000 mg.

At the best of our knowledge our study is the first attempt to model and simulate the tumor growth of non-small cell lung cancer in space and time. We validated our models by comparing the time behavior of the longitudinal size of the tumor ellipsoid with the theoretical and experimental results of Tham et al. [18]: we found a good agreement between the experimental data and the predictions of Model 2 and Model 3, such as the dosage of body gemcitabine necessary to slow down and arrest the growth of tumor (about 10 *μg *of body dose as in [18]), and the rate of tumor growth in the case of an insufficient amount of drug (between 0.5 an 1 mm per week as in the graph reported in [18]). Moreover these models confirm the correlation between glucose uptake and pharmacological treatment as reported by Duhaylongsod et al. in [44]. Namely, comparing the results of Model 2 and Model 3 with those of Model 1 we confirmed the necessity of a higher dose of gemcitabine or conversely of the reduction of the glucose uptake [18,48] for obtaining a significant increment of tumor shrinkage.

## Conclusions

We have presented a computational framework for modeling and simulating the spatial dynamics of the diffusion of biological entities at micro- and meso-scale in a non-homogeneous medium. We use these mathematical and computational structure to model and simulate a non-small cell lung cancer treated with gemcitabine. The drug efficacy and the rate constant of resistance appearance have been estimated from real tumor growth curves recorded in 56 patients. The other parameters have been obtained from the literature reporting the in vitro experiments of the last decade. We explored the behavior of the model under different conditions concerning the rate of glucose uptake, the number of tumor cells and the dose of gemcitabine. The proposed models reproduce the expected tumor growth rate at the optimal body concentration of gemcitabine and confirm the correlation between glucose uptake and the response to the chemotherapy. At the best of our knowledge, this study is the first attempt to build a reaction-diffusion model of non-small cell lung cancer by integrating data from in vivo experiments and by inferring kinetic parameters from the tumor shrinkage curves of patients with the purpose to provide in silico-generated dynamical images of the morphology of this kind of tumor.

Nonlinear models of cancer growth are needed to understand the phenomenon of realistic cancer growth. Simulations of such models conducted to determine the patterns of cancer growth and cancer response to drug and nutrient supply could support the design of the administration schedule and the duration of the therapy. Moreover, a computational model of a reaction-diffusion system taking into account the stochasticity of the interaction between drugs and tumor cells as well as the non-homogeneity of the intra- and inter-cellular medium may be a contribution toward this direction. Further extensions of this study are in progress and consider the opportunity to include immunological and angiogenic factors and interactions to make the current models more accurate, realistic and of greater medical interest.

## Competing interests

The authors declare that they have no competing interests.

## Authors' contributions

Each author contributed to this work in compliance with his/her expertise field. Paola Lecca developed the mathematical model of the stochastic dynamics of a non-homogeneous reaction-diffusion systems. Paola Lecca also designed the in silico experiments and wrote the scripts for the simulations of the tumor growth with Redi. Daniele Morpurgo contributed to the literature referencing of the study, to the calibration of the models, and to the analysis and validation of the results.
